# Division of the genus *Chryseobacterium:* Observation of discontinuities in amino acid identity values, a possible consequence of major extinction events, guides transfer of nine species to the genus *Epilithonimonas*, eleven species to the genus *Kaistella*, and three species to the genus *Halpernia* gen. nov., with description of *Kaistella daneshvariae* sp. nov. and *Epilithonimonas vandammei* sp. nov. derived from clinical specimens

**DOI:** 10.1099/ijsem.0.003935

**Published:** 2020-01-02

**Authors:** Ainsley C. Nicholson, Christopher A. Gulvik, Anne M. Whitney, Ben W. Humrighouse, Melissa E. Bell, Barry Holmes, Arnie G. Steigerwalt, Aaron Villarma, Mili Sheth, Dhwani Batra, Lori A. Rowe, Mark Burroughs, Jessica C. Pryor, Jean-François Bernardet, Celia Hugo, Peter Kämpfer, Jeffrey D. Newman, John R. McQuiston

**Affiliations:** ^1^​ Special Bacteriology Reference Laboratory, Bacterial Special Pathogens Branch, Division of High-Consequence Pathogens and Pathology, Centers for Disease Control and Prevention, Atlanta, Georgia 30333, USA; ^2^​ National Collection of Type Cultures, Health Protection Agency, Colindale, London NW9 5EQ, UK; ^3^​ Division of Scientific Resources, Centers for Disease Control and Prevention, Atlanta, Georgia 30333, USA; ^4^​ Institut National de la Recherche Agronomique, Unité de Virologie et Immunologie Moléculaires, Domaine de Vilvert, Jouy-en-Josas, France; ^5^​ Department of Microbial, Biochemical and Food Biotechnology, University of the Free State, Bloemfontein, South Africa; ^6^​ Institut für Angewandte Mikrobiologie, Universität Giessen, Giessen, Germany; ^7^​ Biology Department, Lycoming College, Williamsport PA 17701, USA

**Keywords:** *Chryseobacterium*, extinction, genus delineation, taxonomy

## Abstract

The genus *Chryseobacterium* in the family *Weeksellaceae* is known to be polyphyletic. Amino acid identity (AAI) values were calculated from whole-genome sequences of species of the genus *Chryseobacterium,* and their distribution was found to be multi-modal. These naturally-occurring non-continuities were leveraged to standardise genus assignment of these species. We speculate that this multi-modal distribution is a consequence of loss of biodiversity during major extinction events, leading to the concept that a bacterial genus corresponds to a set of species that diversified since the Permian extinction. Transfer of nine species (* Chryseobacterium arachidiradicis *, *Chryseobacterium bovis, Chryseobacterium caeni, Chryseobacterium hispanicum, Chryseobacterium hominis, Chryseobacterium hungaricum*﻿,*, Chryseobacterium pallidum* and *Chryseobacterium zeae *) to the genus *Epilithonimonas* and eleven (* Chryseobacterium anthropi *, *Chryseobacterium antarcticum *, *Chryseobacterium carnis *, *Chryseobacterium chaponense *, *Chryseobacterium haifense, Chryseobacterium jeonii*, *Chryseobacterium montanum *, *Chryseobacterium palustre *, *Chryseobacterium solincola *, *Chryseobacterium treverense* and *Chryseobacterium yonginense *) to the genus *Kaistella* is proposed. Two novel species are described: *Kaistella daneshvariae* sp. nov. and *Epilithonimonas vandammei* sp. nov. Evidence is presented to support the assignment of *Planobacterium taklimakanense* to a genus apart from *Chryseobacterium,* to which *Planobacterium salipaludis* comb nov. also belongs. The novel genus *Halpernia* is proposed, to contain the type species *Halpernia frigidisoli* comb. nov., along with *Halpernia humi* comb. nov., and *Halpernia marina* comb. nov.

The history of the genus *Chryseobacterium* is complicated by multiple instances in which species from other genera have been transferred into or removed from the genus. The genus was proposed in 1994 to contain many of the species that were at that time considered to be members of the genus *Flavobacterium* but were dissimilar both phenotypically and genetically to *Flavobacterium aquatile* (type and only strain: ATCC 11947^T^), which is the type species of the genus *Flavobacterium* [1]. In the same report, the genus *Bergeyella* was created to contain the generically misclassified species *Weeksella zoohelcum* [2] and the genus *Empedobacter* was revived to accommodate the species *Empedobacter brevis *, which had been given a variety of names over the years, including ‘*Empedobacter breve*’ [3, 4]. A decade later, the genus *Kaistella* was created with a single species, *Kaistella koreensis *, to accommodate three strains with 16S rRNA sequences quite distant from the type strains of the species of the genus *Chryseobacterium* that had been published at that time [5]. The next year (2005) the genus *Elizabethkingia* was created based on both 16S rRNA gene sequence and phenotypic differences to accommodate the species previously described as *Chryseobacterium meningosepticum* (reclassified as *Elizabethkingia meningoseptica *), as well as the previously unnamed species *Elizabethkingia miricola* [6]. The genus *Sejongia* was created for *Sejongia antarctica* and *Sejongia jeonii* [7], and *Sejongia marina* was added shortly thereafter [8]. The members of the genus *Sejongia* were later recognised as having a 16S rRNA gene sequence similar to those of *Chryseobacterium haifense* and *Chryseobacterium hominis* and were transferred to the genus *Chryseobacterium* [9] in the same year that the single species in the genus *Kaistella* (* Kaistella koreensis *) was reclassified as a member of the genus *Chryseobacterium* [10]. The genus *Planobacterium* was created with the description of *Planobacterium taklimakanense* [11], which was later proposed to belong to the genus *Chryseobacterium* on the basis of 16S rRNA gene sequence similarity [12]. The genus *Epilithonimonas* was created to accommodate the species *Epilithonimonas tenax* [13], and by 2015 the genus had grown to contain four more species [14–17]. The species of the genus *Epilithonimonas* were subsequently transferred to the genus *Chryseobacterium* [18], despite the recognition that this would cause the genus *Chryseobacterium* to contain three separate lineages. This was considered to be the more conservative reclassification approach as it would result in minimal changes.

There are several genera the members of which are phenotypically similar to members of the genus *Chryseobacterium* but have historically been considered distinct. In 1986, the genus *Weeksella* was created with a single species (* Weeksella virosa *) to contain strains previously named as ‘CDC group IIf’ from human clinical specimens [19]. After the 1994 transfer of *Weeksella zoohelcum* to the genus *Bergeyella *, it remained a single-species genus until 2015, when *Weeksella massiliensis* was described [20]. The genus *Riemerella* was created to accommodate *Riemerella anatipestifer *, named to honour O.V. Riemer, who in 1904 first described the disease *septicemia anserum exsudativa* in geese infected by *Riemerella anatipestifer* [21]. A related bacterium causing respiratory infections in pigeons was later described as *Riemerella columbina* [22]. This species also causes illness in ostriches [23]. *Riemerella columbipharyngis* was found in apparently healthy pigeons [24]. The novel genus and species *Wautersiella falsenii* were created to accommodate clinical isolates that were similar phenotypically to members of the genera *Chryseobacterium* and *Empedobacter* [25], but it was later proposed that the single species in this genus be incorporated into the genus *Empedobacter* [26]. The genera *Soonwooa* [27], *Cruoricaptor* [28] and *Daejeonia* [29] were each established to contain a single species. The genus *Chryseobacterium* and all of these related genera had been previously described as belonging to the *Chryseobacterium/Riemerella* branch of the family *Flavobacteriaceae* but results from a whole genome sequence (WGS) analysis of 1000 type strain genomes from the phylum *Bacteroidetes* [30] recently indicated that they are distinct from the *Flavobacteriaceae,* and the family name *Weeksellaceae* was proposed for them.

WGS analysis has revolutionised the identification and taxonomic classification of bacterial isolates. High-coverage contigs from draft genome assemblies of short sequence reads (e.g., Illumina, with at least 50× coverage) have been shown to produce results indistinguishable from those produced using complete circularised genomes when values such as the average nucleotide identity using blast (ANIb) and genome-to-genome distance calculation (GGDC) formula 2 predicted DNA–DNA hybridization (DDH) are calculated [31–33]. Consequently, the WGS analyses described here are based on high-coverage draft genomes. The ANIb 95 % cut-off value for species delimitation [34] was calculated to be equivalent to a GGDC-predicted DDH value of slightly less than 65%, lower than the DDH value of approximately 70 % which has long been used for species delimitation [32]. This might support the 96.5 % whole-genome ANI (gANI) species threshold that has been suggested [35], or it might simply be an artefact of the inexactness of traditional DDH measurements. The use of WGS data in the naming of species is supplanting 16S rRNA sequence analysis [36], and some have argued for allowing the naming of a species based solely on WGS data, with the genome assembly itself serving as the type material [37].

A set of 182 isolates (primarily of clinical origin) belonging to, or resembling phenotypically, the members of the genera *Chryseobacterium *, *Elizabethkingia* and *Empedobacter* was previously analysed using DNA–DNA hybridization and comparative 16S rRNA gene sequence analysis, resulting in the description of four novel species that each contained between two and six strains. The majority of the other isolates could be assigned to already-described species, but 15 strains each appeared distinct from all others and almost certainly represented novel species [12]. We refrained from proposing names for these, as per recommendations that multiple strains be used in describing a species [38]. The current study continues that previous work with a comprehensive whole-genome sequence analysis of those isolates. Over the course of this collaboration we have added 19 complete type strain genomes to the 85 genomes attributed to the type strains of species of the genus *Chryseobacterium* that were previously available and 20 additional genomes, some of which are the sole representative of yet-unnamed species. In the process we compared these to other species from the genus *Chryseobacterium* and its nearest neighbours, specifically those which were characterised in the Bergey’s chapter on *Chryseobacterium* and related genera [39] and came to the conclusion that a taxonomic realignment was necessary. Distribution of AAI values calculated from type strain comparisons was found to be multimodal, which led us to propose a re-organization based on average amino acid identity (AAI) that separates the *Chryseobacterium* genus into four different genera. These correspond to the multiple lineages apparent in previous publications [18] and also recognised as separate genera in the Genome Taxonomy Database [40]. This aligns genus assignments so that the type strain for each species in each genus has ≥76 % AAI values compared with the type strain of the type species of its genus.

Twenty-eight strains whose genome sequence has not previously been reported are listed in Table 1. This includes the type strains of *Chryseobacterium lactis *, *Chryseobacterium carnis, Chryseobacterium bernardetii* and *Chryseobacterium nakagawai* that had been described in the earlier study [12], along with one or two additional strains from the same DNA–DNA hybridization group. Of the DNA–DNA hybridization groups that were assigned in that paper to existing species (based on 16S rRNA gene sequence), a representative strain was selected for sequencing. Additional strains that might be taxonomically informative were identified by reviewing records of 16S rRNA gene sequences from isolates in the Special Bacteriology Reference Laboratory (SBRL) strain collection. Several members of the DNA–DNA hybridization group that we believed to be *Chryseobacterium taklimakanense *, additional representatives of several species of the genus *Chryseobacterium* that were well-represented in the collection, and strains of several suspected novel species were selected for sequencing. These 28 genomes were compared with genomes available in the public domain, including genomes from the type strains of all species that were considered to belong to the genus *Chryseobacterium* (*n*=76), genomes of selected strains considered to be members of the genus *Chryseobacterium* and representative of their respective species, but not type strains (*n*=4), and genomes of the type strains of species from the ‘nearest neighbour’ genera discussed in the introduction (*n*=20) (Table S1, available in the online version of this article). There remain more than 30 species of the genus *Chryseobacterium* with validly published names that have no type strain genome sequenced.

**Table 1.** Details on newly sequenced strains tDDH, traditional DNA–DNA hybridization; nd, not determined.

Strain number sequenced	Other strain numbers	Previously described as:	Proposed as:	Geographic location	Source	Date isolated	tDDH group	DDH_#	Accession Number
G0081	CL88/78; Hayes B19/1	* Chryseobacterium carnis *	*Kaistella carnis*	Unknown	Beef	1973	95	95	CP034159
F5649	CCUG 73498, CIP 111693	* Chryseobacterium hominis *	*Epilithonimonas vandammei*	Iowa, USA	Testicle	1984	224	224	CP034161
H6466	CCUG 73281	* Chryseobacterium hominis *	*Epilithonimonas vandammei*	Indianapolis, Indiana, USA	Leg wound	July, 2013	nd		CP034160
H3001	CCUG 73276; CIP 111694	Unidentified	*Kaistella daneshvariae*	New York, USA	Peritoneal cavity	January, 2004	nd		CP034158
F9257	CL712/92	* Chryseobacterium taklimakanense *	* Planobacterium taklimakanense *	Florida, USA	Blood	1987	212	212	CP034173
H4753	CCUG 73278	* Chryseobacterium taklimakanense *	* Planobacterium taklimakanense *	Michigan, USA	Blood	2008	nd		CP034171–CP034172
H5297	NCTC 13453; CCUG 52711; CIP 109415; DSM 22165; NF802	* Chryseobacterium hominis *	*Epilithonimonas hominis*	La Louvière, Belgium	Blood	1998	nd		RJTU00000000
G0240	CL278/82; CCUG 73500	‘123 group’	Chryseobacterium sp. nov.	Darlington, England	Urine	1982		153	RJTV00000000
G0235	CL542/79; CCUG 73274	‘71 group’	* Chryseobacterium cucumeris *	London, England	Green lizard	1979	71	148	RJTW00000000
H4373	KC_2159; CPW406; KCTC 12088; NBRC 102008; DSM 15235	* Chryseobacterium daecheongense *	* Chryseobacterium daecheongense *	Lake Daecheong, Korea	Freshwater lake sediment		nd		RJTX00000000
F4391	CL720/92	* Chryseobacterium anthropi *	*Kaistella haifensis*	Indiana, USA	Lung	1983		236	RJTY00000000
H3056	CCUG 73499	Unidentified	*Kaistella daneshvariae*	New Mexico, USA	Blood	2004	nd		RJUG00000000
G0079	CL311/80; A16/80; CCUG 73267	* Chryseobacterium anthropi *	*Kaistella haifensis*	Midlothian, Scotland	Calf	1980	93	93	CP033933
G0229	CL318/82	* Chryseobacterium bernardetii *	* Chryseobacterium bernardetii *	Doncaster, England	Sputum	1982	142	142	CP033932
G0211	CL97/78; Hayes S10/1; CCUG 73273	* Chryseobacterium indoltheticum *	* Chryseobacterium indoltheticum *	Unknown	Soil	Unknown	* C. indoltheticum *	140	CP033928
G0197	A139/68; CCUG 73271	* Chryseobacterium lactis *	* Chryseobacterium lactis *	Paisley, Scotland	Milk bottle rinse	Unknown	63	128	CP033925
KC_1864	NCTC 11390, A140/68, F68	* Chryseobacterium lactis *	* Chryseobacterium lactis *	Paisley, Scotland	Milk bottle rinse,	Unknown	63	63	CP033924
G0041	F91	* Chryseobacterium nakagawai *	* Chryseobacterium nakagawai *	Gloucester, England	Kidney abscess	Late 1960's	78	78	CP033923
G0162	CL192/74; CCUG 73268	‘78 group’	* Chryseobacterium* sp. nov.	Newcastle-upon-Tyne, England	Urine	1974	78	117	CP033922
F9942	A103/68; CCUG 73266	‘125 group’	* Chryseobacterium carnipullorum *	Paisley, Scotland	Milk swab	Unknown	125	64	CP033921
G0188	A104/68; CCUG 73270	‘125 group’	* Chryseobacterium carnipullorum *	Paisley, Scotland	Milk swab	Unknown	125	125	CP033920
G0186	A86/68; CCUG 73269	‘123 group’	* Chryseobacterium* sp. nov.	London, England	Human	Unknown	123	123	CP033918–CP033919
G0201	CL381/78	‘132 group’	* Chryseobacterium* sp. nov.	Dublin, Irish Republic	Drinking water	1978	132	132	CP033917
G0207	CL189/78; CCUG 73272	‘132 group’	* Chryseobacterium shandongense *	Nuneaton, England	Zimmer water bag	1978	132	136	CP033915–CP033916
G0239	CL141/82; CCUG 73275	‘132 group’	* Chryseobacterium shandongense *	London, England	Cryoprecipitate, transfusion reaction	1982	nd	152	CP033914
H4638	CCUG 73277	* Chryseobacterium bernardetii *	* Chryseobacterium bernardetii *	Pennsylvania, USA	Larynx	2008	nd		CP033931
H5559	CCUG 73280	* Chryseobacterium indologenes *	* Chryseobacterium indologenes *	Wailuku, Hawaii, USA	Blood	2010	nd		CP033930
H5143	CCUG 73279	‘132 group’	* Chryseobacterium shandongense *	Tennessee, USA	Cerebrospinal fluid (CSF)	2009	nd		CP033912–CP033913

DNA–DNA hybridization was performed as described previously [41]. All strains were biochemically characterised in all or most of a range of 68 conventional biochemical tests by methods described previously [42] and whole-genome sequences were generated as described previously [32, 43–47].

Sequence alignments were performed using the ClustalW module in BioEdit v7.0.5.3 [48]. For analysis of 16S rRNA genes, sequences were trimmed to match the start and end of the near full-length sequence JX100817 (deposited as *Chryseobacterium carnis* strain G0081^T^). All pairwise comparisons with a 16S rRNA gene sequence identity greater than >98.65 % were considered as potentially representing the same species, as this limit has been recommended for species demarcation [49]. The *rpoB* gene sequences were extracted from WGS data as described previously [32]. Phylogenetic trees were reconstructed using MEGAX [50] with the Jukes-Cantor substitution model.

Proteomes from each genome were generated by Prodigal v2.6.2 [51]. For each pairwise comparison, an all-versus-all search of all proteins was carried out using BLASTp v2.4.0+ [52] in both directions. If both directions of BLASTp searches resulted in the same protein match (pair) and exceeded 40 % in amino acid identity and 50 % in coverage length, we included the protein sequences for computing the arithmetic mean amino acid sequence identity (AAI). Percentage of conserved proteins (POCP) scores were calculated as previously described [53]. GGDC formula 2 was used to generate the predicted DDH values. The Jspecies software package version 1.2.1 was used to calculate average nucleotide identity with BLASTn alignments (ANIb) [31, 34]. Additional data visualizations were produced using JMP v11 (SAS Institute).

The gene pairings identified during the AAI pairwise comparisons described above were used to generate a list of 488 loci present in a single copy in all of the strains listed in Table S1. The maximum sequence length for each orthologous group was computed, and groups with members having a length ≥90 % of this maximum were selected for alignment. These 148 were aligned (as protein sequences) using ClustalW in BioEdit v7.0.5.3 [48], and manual refinements were made to align start and stop codons. Unusually divergent (<25 % nucleotide identity) loci were filtered out, and the remaining 119 gene sequences from each genome were concatenated. The most highly conserved of these loci were selected using a criterion that they had to have at least 25 % identity at the nucleic acid level. Gaps and invariant positions were masked, which left 68,272 core variable nucleotide sites. Maximum-likelihood analysis using the Jukes-Cantor substitution model was performed in RAxML ver 8.2.12 [54]. The best scoring topology of 250 maximum-likelihood trees had 100 bootstrap replicates overlaid. The extended majority-rule consensus indicated convergence (1.54 % weighted Robinson–Foulds mean) after 100 replicates [55].

Historically, there have been no unambiguous criteria for creation of a novel genus, and mis-classification of species has occurred frequently. The addition of 16S rRNA gene sequence analysis to the phenotypic characterization of strains, which was originally used for taxonomic classification, represented a marked improvement but for several reasons is not ideal. Firstly, 16S rRNA gene sequences can differ at their various loci within a strain’s genome. This has been observed in the genus *Elizabethkingia* [32] as well as the genera *Pseudomonas* [56], *Prevotella* [57] and *Neiserria* [58]. Secondly, while a 16S rRNA gene sequence similarity below 98.5 % can ensure that two organisms will have less than 70 % DDH and therefore be separate species [59], the converse is not true. Organisms may have 16S rRNA gene sequences over 99 % similar or even identical and still belong to different species, with DDH values below 70 % and ANI values less than 95 %. For example, the type strains of *Chryseobacterium shigense* [60] and *Chryseobacterium carnipullorum* [61] share 99.3 % identity in their 16S rRNA gene sequences, which could indicate they belong to the same species, but genome comparisons suggest otherwise (DNA–DNA hybridization <44 % [62, 63] and ANIb <91 %). The 16S rRNA gene sequences of *Chryseobacterium jejuense* are also very similar to those of *Chryseobacterium nakagawai* and *Chryseobacterium lactis* (98.9 and 98.8% identical, respectively), but the ANIb values from genome comparisons (averaging <87 and<82 %, respectively) confirm that all are separate species.

There are several species that have been included in the genus *Chryseobacterium* on the basis of the results of 16S rRNA gene sequence analysis whose inclusion is not supported by the additional evidence of whole-genome sequence and/or reconstruction of a phylogenetic tree containing sequences from all of the closely related species. The proposal to include *Planobacterium taklimakanense* within the genus *Chryseobacterium* was made based on a 16S rRNA phylogenetic tree [12], and consequently the species *Chryseobacterium frigidum* was included in the genus *Chryseobacterium* in large part due to its similarity with *Chryseobacterium taklimakanense* [64], but an analysis that included all of the species of the genus *Chryseobacterium* and additional species of the family *Weeksellaceae* (Fig. S1), indicates it is more closely related to the members of the genus *Cruoricaptor *. The 16S rRNA gene sequence for the effectively but not yet validly published species ‘*Chryseobacterium chengduensis’* is consistent with it being outside the genus *Chryseobacterium* (possibly in the genus *Daejeonia *) when all of the closely related species are included. The assignment of this species to the genus *Chryseobacterium* was made based on an incomplete analysis that lacked an outgroup and included only 18 species of the genus *Chryseobacterium *, including several that we classify as belonging to the genus *Kaistella* [65]. The species *Chryseobacterium reticulitermitis* was described as a member of the genus *Chryseobacterium* based on a neighbour-joining tree that used the distantly related species *Gramella echinicola* as the outgroup. Our more extensive neighbour-joining tree (Fig. S1) located the 16S rRNA gene sequence of *Chryseobacterium reticulitermitis* near to the base of the tree. To include it as a member of the genus *Chryseobacterium* would require the undesirable inclusion of other well-established genera, such as *Riemerella* and *Cloacibacterium,* into the genus *Chryseobacterium* also. It is clear that genus assignment based on 16S rRNA gene sequencing has serious limitations, and that an objective and reproducible method for genus determination is needed.

Initially, a proposed method for genus delineation based on Percentage of Conserved Proteins (POCP) [53] was investigated for its utility in distinguishing species belonging to the genus *Chryseobacterium* and related genera. Calculation of AAI values based on the specified parameters was an intermediate step in generating the protein comparisons for POCP, but we found AAI values themselves to be more informative than POCP. This was fortunate, as the use of AAI values for genus determination has inherent advantages over POCP, particularly for incomplete genomes. Reciprocal AAI values are limited to the range of 0 to 100 regardless of protein quantities in each genome and are symmetric, so completeness of the genome will have less of an effect on this metric.

When AAI was calculated for type strain comparisons between species of the genus *Chryseobacterium* and species from related genera using the parameters specified for POCP, all AAI values were >65 %, and the distribution was bi-modal (Fig. 1). Out of 8646 AAI comparisons, only 44 (0.5 %) were between 74 and 76 %. This suggested a natural cut-off value between 74 and 76 %. Comparisons using complete genomes produced the same AAI results as when draft genomes were used (data not shown), which was expected for genomes that meet or exceed the minimum standards [66]. Most comparisons between strains that had an AAI of ≥76 % were categorised as representing members of the same genus, while most that had an AAI≤74 % represented members of different genera. Of the strain comparisons that deviated from this general rule, many contained strains that had been originally categorised as belonging to one of the genera that were later subsumed into the genus *Chryseobacterium *. We have formulated a strategy of genus delineation based on the criterion that the type strain of a species must have an AAI value greater than 76 % when compared with the type strain of the type species of its genus in order to represent a member of the same genus, and that all type strains must have an AAI value greater than 74 % when compared with each other. Exact AAI values calculated are parameter-dependant, so any application of this 74–76 % cut-off for genus delineation must use AAI values calculated with the parameters of 40 % amino acid identity and 50 % coverage length. The discontinuities of AAI distributions used to realign the polyphyletic genus *Chryseobacterium* were thus used in a manner similar to taxonomic redistributions of the genera *Rhodococcus* [67] and *Mycobacterium* [68].

**Fig. 1.**
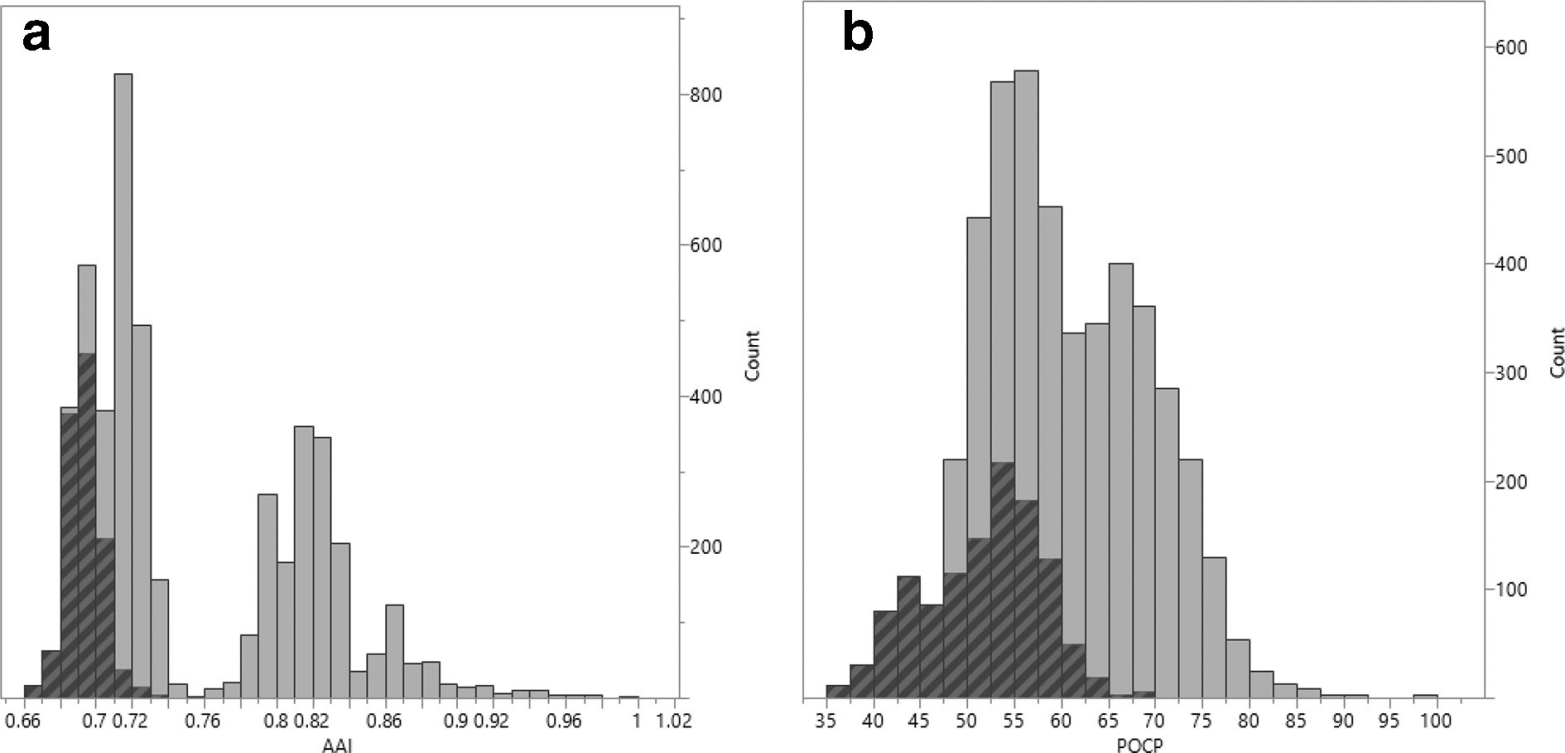
Distribution of (A) AAI% and (B) POCP, for type strain comparisons where both strains are listed in LSPN as members of the genus *Chryseobacterium* (which includes the strains described in this paper as members of the genera *Epilithonimonas *, *Halpernia*, *Kaistella* or *Planobacterium *), or one of its closest relatives (* Bergeyella *, *Cloacibacterium *, *Cruoricaptor *, *Elizabethkingia *, *Riemerella* ﻿or *Soonwooa *). Dark grey indicates comparisons between strains already considered to belong to different genera. Note that few comparisons yield an AAI between 74 and 76 %, and that unlike POCP distribution, AAI distribution is bi-modal.

Just like the distribution of AAI values, the distribution of ANI values is discontinuous [69]. A dearth of ANI values in the low 70 % s among *Enterococci* [70] was attributed to the Permian extinction approximately 252 million years ago (MYA) which is believed to have eliminated 90–95 % of all species on the planet [71, 72]. The Permian was the third of five recognised major extinction events in the history of our planet [73–76]. A major extinction is characterised by a loss of biodiversity, followed by a re-diversification of the survivors [77]. This process pertains not only to multi-cellular fossil-forming organisms, but also to organisms for which there is no good fossil record, as evidenced by studies of microbialites [78] and of lichen-forming fungi [79]. In order to assess whether the observed AAI gap between 74 and 76% among members of the family *Weeksellaceae* would correspond to this gap in ANI values due to the Permian extinction, we charted AAI data for each strain comparison against average nucleotide identity blastn (ANIb) data for the same set of strains (data available on request), and found that a quadratic fit could be used to derive a formula that calculates the expected AAI from the ANIb (Fig. S2, part A). The gap in AAI values from 74 to 76 % does indeed correspond to an expected gap in ANIb values between 72.5 and 74 %, and examination of the distribution of ANIb scores shows a similar gap in this range (Fig. S2, part B).

We reasoned that if the gap in AAI values between 74 and 76 % represented the Permian extinction, then the second, less distinct valley in the distribution histogram that appears at an ANIb value of approximately 80 % might be an artefact of the Triassic extinction, which ended approximately 201 MYA [80]. Linear regression using these values, and a value of 100 % ANI for the present time, predicts that divergence of strains with the 95 % ANI that is used for species delineation [34] would have occurred approximately 65 MYA. This coincides with the timing of the Cretaceous–Paleogene (K-pg) extinction (notable for elimination of the non-avian dinosaurs [81–83]). If our calculations are correct that would mean that a bacterial species has a common ancestor which survived the K-pg extinction, and we could think of a genus as having a common ancestor that survived the Permian extinction (Fig. S3).

None of this actually proves our speculation that the multi-modal distribution of AAI (and ANIb) observed when large numbers of strain comparisons are examined is a consequence of extinction events. Regardless of their etiology, these naturally-occurring ‘gaps’ in distribution of AAI values can be utilised in a standardised taxonomic strategy [67].

In proposing to subdivide the genus *Chryseobacterium *, we contradict an earlier proposal [18] to merge these genera despite the evidence from whole-genome sequence data that doing so would result in a polyphyletic genus. The preference to reduce the number of taxonomic changes in the absence of a reliable method of genus delineation was reasonable at the time. Our strategy of genus delineation based on AAI values was developed based on the observation that strain comparisons yielding AAI values between these cut-off points are rare, and we suggest that this rarity is due to the likelihood that most of the bacterial cells whose descendants would have been separated by AAI values in this range were instead eliminated in the Permian extinction. Using this criterion places a number of species into the genera *Kaistella* (including several originally classified as members of the genus *Sejongia *)*, Epilithonimonas* or *Planobacterium* instead of the genus *Chryseobacterium,* and it reveals a previously unrecognised genus containing three of the species. The core genome analysis of these species, shown in Fig. 2, supports these divisions, and they can be readily distinguished by *rpoB* sequence analysis (Fig. S4).

**Fig. 2.**
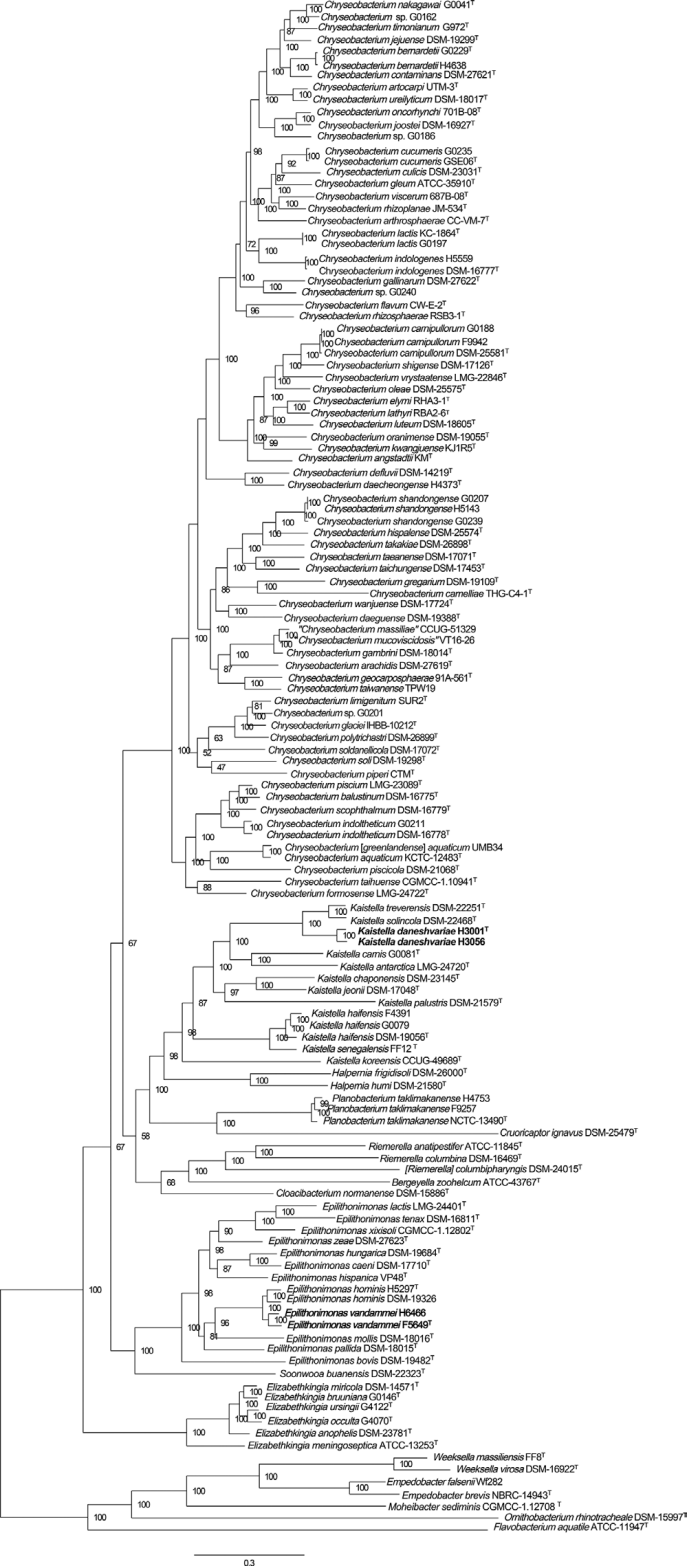
Maximum likelihood phylogenetic analysis of core genome loci from the members of the genus *Chryseobacterium* and closely related genera. The scale bar indicates substitutions per core variable site (*n*=68,272).

The newly-sequenced strains NCTC 13454^T^ (* Chryseobacterium joostei *), NCTC 10796^T^ (* Chryseobacterium indologenes *), and NCTC 11390^T^ (* Chryseobacterium lactis *) were confirmed as members of the genus *Chryseobacterium* based on AAI values of >76 % when compared with strain ATCC 35910^T^ of *Chryseobacterium gleum,* the type species of the genus *Chryseobacterium *. In contrast, AAI values comparing the type strain of *Epilithonimonas tenax* (the type species of the genus *Epilithonimonas *) to other strains originally described as representing members of the genus *Epilithonimonas* were all >76 %, and those strains all have AAI values >76 % when compared with each other, but <74 % (range 71.34–71.97 %, mean=71.70 %) when compared with *Chryseobacterium gleum* ATCC 35910^T^. Similarly, most strains that had originally been described as members of the genera *Sejongia* or *Kaistella* had AAI values >76 % (mean=80 %) when compared with each other or with the strain that was originally described as *Kaistella koreensis,* but <74 % (mean=72 %) when compared with *Chryseobacterium gleum* ATCC 35910^T^.

The genus *Epilithonimonas* would thus comprise all its original species (* Epilithonimonas lactis *, *Epilithonimonas ginsengisoli *, *Epilithonimonas psychrotolerans* and *Epilithonimonas xixisoli *, and *Epilithonimonas tenax* as the type species) with the addition of *Chryseobacterium arachidiradicis* renamed as *Epilithonimonas arachidiradicis* comb. nov.*, Chryseobacterium bovis* as *Epilithonimonas bovis* comb. nov.*, Chryseobacterium caeni* as *Epilithonimonas caeni* comb. nov.*, Chryseobacterium hispanicum* as *Epilithonimonas hispanica* comb. nov.*, Chryseobacterium hominis* as *Epilithonimonas hominis* comb. nov.*, Chryseobacterium hungaricum* as *Epilithonimonas hungarica* comb. nov.*, Chryseobacterium molle* as *Epilithonimonas mollis* comb. nov.*, Chryseobacterium pallidum* as *Epilithonimonas pallida* comb. nov. and *Chryseobacterium zeae* as *Epilithonimonas zeae* comb. nov. The genus *Kaistella* would consist of its original species (* Kaistella koreensis *) as the type species, and the species *Chryseobacterium antarcticum* (formerly the type species of the genus *Sejongia *) would be named as *Kaistella antarctica* comb. nov., *Chryseobacterium carnis* as *Kaistella carnis* comb. nov., *Chryseobacterium chaponense* as *Kaistella chaponensis* comb. nov., *Chryseobacterium haifense* as *Kaistella haifensis* comb. nov.*, Chryseobacterium jeonii* as *Kaistella jeonii* comb. nov.*, Chryseobacterium montanum* as *Kaistella montana* comb. nov.*, Chryseobacterium palustre* as *Kaistella palustris* comb. nov.*, Chryseobacterium solincola* as *Kaistella solincola* comb. nov.*, Chryseobacterium treverense* as *Kaistella treverensis* comb. nov.*,* and *Chryseobacterium yonginense* as *Kaistella yonginensis* comb. nov. The effectively published *Chryseobacterium senegalense*, which has not yet been added to the validation lists, would also be placed into the genus *Kaistella *.

Two species do not represent members of any of the genera proposed above, but instead have AAI values indicating that they comprise a separate novel genus. We propose to name this genus after Dr. Malka Halpern, Professor at University of Haifa in Israel. Consequently, the novel genus *Halpernia* contains *Chryseobacterium frigidisoli* renamed as the type species *Halpernia frigidisoli* comb. nov., and *Chryseobacterium humi* renamed as *Halpernia humi* comb. nov. On the basis of the results of 16S rRNA sequence analysis (Fig. S1), it appears that strain IMCC3228, originally designated as *Sejongia marina* and later as *Chryseobacterium marinum,* also represents a member of this genus, so we propose to rename it as *Halpernia marina* comb. nov.

This study is an extension of DNA–DNA hybridization studies performed on NCTC and CDC strains which were originally named as belonging to CDC groups IIc, IIe, IIh and IIi [84]. While many of these strains had been described as representing novel species or assigned to already-described species [12], other DNA–DNA hybridization groups could not be assigned for various reasons. WGS analysis was applied to resolve these lingering questions.

Strain G0235 can be assigned to the species *Chryseobacterium cucumeris* as its genome has an ANIb of 98.41 % when compared with the type strain of that species. It is the only one of the ‘71 group’ strains for which we have a sequenced genome, but the *rpoB* gene was sequenced for strains F9971 and F9973. The gene sequences for all three strains clustered with the *rpoB* sequence for the type strain of *Chryseobacterium cucumeris* (see Fig. S4). The ‘71 group’ can therefore be considered to be *Chryseobacterium cucumeris *.

G0079, the representative strain of the ‘93 group’, had been attributed to *Chryseobacterium anthropi* on the basis of its 16S rRNA gene sequence [12], but its whole-genome sequence revealed that it actually belongs to the species *Kaistella haifensis* based on ANIb similarity of 95.56 % with *Kaistella haifensis* strain DSM19056^T^. *Kaistella haifensis* can be included among the clinically relevant species, as many of the ‘93 group’ members were derived from human clinical specimens. There are currently no available sequence data for the type strain of *Chryseobacterium anthropi,* CCUG 52764, but we do have whole genome sequence data for our strain F4391 (=CCUG 15260) which was used in the species description for *Chryseobacterium anthropi *. Strain F4391 was also confirmed to be a strain of *Kaistella haifensis* by ANIb results of 96.14 %/96.18% and 95.68 %/95.50%, when compared with strains G0079 and DSM 19056^T^, respectively. *Chryseobacterium anthropi* was described as distinguishable from *Chryseobacterium haifense* [85] by a lack of acid production from fructose, lactose and sucrose and by a negative ONPG test (β-galactosidase) [10], all of which were positive for *Chryseobacterium haifense* DSM 19056^T^. All ‘93 group’ strains are negative for β-galactosidase, and for production of acid from lactose and sucrose; most, however, produce acid from fructose. Taken together, these findings cast doubt on whether *Chryseobacterium anthropi* is actually a separate species. As this question cannot be answered definitively without a genome sequence of the type strain of *Chryseobacterium anthropi *, we propose the name *Kaistella anthropi* to accommodate strain CCUG 52764.

Strains F9942 and G0188 belonged to the ‘125 group’, which was assigned to *Chryseobacterium shigense* on the basis of 16S rRNA gene sequence similarity and a DDH value of 77 % at 55 °C [12]. 16S rRNA gene sequence analysis does not distinguish between *Chryseobacterium shigense* and the later-named *Chryseobacterium carnipullorum *, but the paper describing *Chryseobacterium carnipullorum* found the two species to differ by a DDH value of 56.1 % [61]. The ANIb value of strains F9942 and G0188 when compared with each other was 99.98%, and when compared with the type strain of *Chryseobacterium carnipullorum* were 98.17 and 97.96 %, respectively. The ANIb values of any of these strains when compared with the type strain of *Chryseobacterium shigense* were less than 91 %. Thus, members of the ‘125 group’ do not belong to *Chryseobacterium shigense,* instead they belong to the species *Chryseobacterium carnipullorum *. The two species are readily distinguished by *rpoB* gene sequence.

The results of our previous 16S rRNA gene sequence analysis indicated that strains G0201 and G0207 represented different species despite being both assigned to the ‘132 group’ in DNA–DNA hybridization studies. The 16S rRNA sequence of G0201 is most similar to that of *Chryseobacterium polytrichastri *, while the 16S rRNA sequences of strains G0207, G0239, and H5143 are most similar to that of *Chryseobacterium shandongense *, differing at a single nucleotide. Long-read (PacBio) sequencing was done on all four strains. ANIb comparisons using these assemblies confirms that strains G0207, G0239, and H5143 represent a single species, and differ from G0201 and any other species whose genome has been sequenced thus far, which would be expected as the type strain of *Chryseobacterium shandongense* has not yet been sequenced. There are phenotypic differences between the type strain of *Chryseobacterium shandongense* and the sequenced ‘132 group’ strains G0207, G0239, and H5143; the latter all grow on MacConkey agar, and none are mucoid. The species description [86] is emended to include the phenotypes of *Chryseobacterium shandongense* strains from our ‘132 group’.

The ‘224 group’, represented by strain F5649, was previously attributed to *Chryseobacterium hominis* on the basis of 16S rRNA gene sequence analysis and DNA–DNA hybridization data [12]. Further confirmation of this species assignment was sought by sequencing the original strain F5649, and a modern isolate (H6466) that had been reported by our lab as *Chryseobacterium hominis *. Strains F5649 and H5466 represented the same species, as expected. Their genomes were compared with *Chryseobacterium hominis* strains NCTC 13453^T^ and DSM 19326. The latter was the type strain of ‘* Chryseobacterium arothri *’, a validly published species later determined by 16S rRNA sequence analysis, DNA–DNA hybridization, and phenotypic similarity to represent a member of *Chryseobacterium hominis* [87, 88]. NCTC 13453^T^ has an ANIb >95 % when compared with *Chryseobacterium* DSM 19326, providing confirmation that *‘Chryseobacterium arothri’* is indeed a junior synonym for *Chryseobacterium hominis *, but both strains had an ANIb <94 % when compared with ‘224 group’ strains. The *rpoB* gene sequence was not useful in distinguishing among the four strains, as the *rpoB* sequence of *Epilithonimonas hominis* NCTC 13453^T^ was 96.5 % identical with that of the other *Epilithonimonas hominis* strain (DSM 19326), but comparisons between *Epilithonimonas hominis* strains and '224 group' strains ranged from 95.4 to 98 %. The ‘224 group’ strains differed from *Chryseobacterium hominis* in that they did not produce a DNase and did not produce acid from salicin. Characteristics that were consistently positive or negative are listed in the species description, but there was considerable variability among the strains. Most strains gave positive results (those giving negative results are given in parentheses) for acid production (in ammonium salt medium) from glycerol (CL263/70, CL205/78, CL213/83), casein digestion (CL205/78), gelatinase production (stab method: CL205/78, CL213/83; plate method: CL205/78), hydrolysis of starch (CL263/70, CL184/75, CL187/75, CL205/78), of Tween 20 (CL205/78) and of Tween 80 (CL263/70, CL195/76, CL205/78, CL373/79, CL213/83) and were oxidative according to the Hugh and Leifson O-F test (CL195/76).

Most strains gave negative results (those giving positive results are shown in parentheses) for acid production (in ammonium salt medium) from ethanol (CL309/73, CL184/75, CL187/75, CL205/78, CL445/80), fructose (CL263/70, CL195/76, CL373/79, CL445/80), mannitol (CL263/70), rhamnose (CL263/70) and sucrose (CL184/75, CL187/75, CL205/78, CL445/80), from glucose in peptone water medium (CL195/76, CL205/78) and from 10 % (w/v) glucose (CL445/80, CL213/83), growth on MacConkey agar (CL195/76, CL205/78, CL373/79), nitrate reduction (CL205/78, CL373/79, CL213/83), nitrite reduction (CL373/79, CL445/80), production of extracellular deoxyribonuclease (CL263/70, CL309/73, CL524/73, CL195/76) and of a brown melanin-like pigment on tyrosine agar (CL373/79).

On the basis of both its phenotypic differences from *Chryseobacterium hominis *, and whole genome sequence results, the ‘224 group’ thus represents a novel species which we propose to name *Epilithonimonas vandammei* after Peter Vandamme, first author on the paper that originally described *Chryseobacterium* as a novel genus.

Strain G0162 was one of two strains originally assigned to the ‘78 group’ (the representative of this group was G0041, later designated as the type strain of *Chryseobacterium nakagawai *). The assignment of G0162 to the ‘78 group’ was marginal as its hybridization was only 69 % at 55 °C; its ANIb of 94 % compared with G0041 indicates that it does not represent a member of the same species. Its 16S rRNA sequence is most similar to that of the type strain of *Chryseobacterium rhizoplanae* JM-534^T^, but ANIb comparisons between this strain’s genome and those of assemblies of two *Chryseobacterium rhizoplanae* SRA data sets (SRX3054699 and SRX3054698) were in the range of 81–82 % (data not shown). Thus G0162 is currently the only known representative of its species. The description of *Chryseobacterium nakagawai* is emended below to reflect removal of strain G0162 from the species.

Strain G0186 was the representative strain for the ‘123 group’, along with strain G0240 and was identified as *Chryseobacterium ureilyticum* on the basis of a 16S rRNA gene sequence identity of 99.2 % [12]. However, its ANIb compared with *Chryseobacterium ureilyticum* strain DSM 18017^T^ was less than 83 % and it did not match any other species. Furthermore, strain G0240 was found to have an ANIb <86 % when compared with all other strains of members of the genus *Chryseobacterium *, including G0186. Hence, the ‘123 group’ is not a group at all, and each isolate is currently the sole representative of its species.

Two strains in the SBRL collection, H3001 and H3056, had identical 16S rRNA gene sequences that were also a close match with the 16S rRNA gene sequences from the type strains of *Chryseobacterium treverensis* (99.3%) and *Chryseobacterium solincola* (99.0%), both of which are proposed herein to represent members of the genus *Kaistella *. Their ANIb results of >96 % when compared with each other, but between 86.6 and 86.94% when compared with *Chryseobacterium treverense* or *Chryseobacterium solincola,* indicate that they represent a novel species. We propose below the name *Kaistella daneshvariae* for this species, in recognition of Maryam Daneshvar. Dr. Daneshvar led the SBRL team during a phase of extraordinary productivity that culminated in the 1996 publication of the second edition of the Manual for Identification of Unusual Pathogenic Gram-Negative Aerobic and Facultatively Anaerobic Bacteria.

In 2013, we proposed to move the species *Planobacterium taklimakanense* into the genus *Chryseobacterium *, primarily on the basis of its 16S rRNA gene sequence [12]. On the basis of the WGS information now available, we consider our original proposal to have been premature. Since the 16S rRNA gene from *Chryseobacterium salipaludis* JC490^T^ clusters with the *Planobacterium taklimakanense* 16S rRNA gene sequences (Fig. S1)but has sequence identity <95 %, this species is probably a second species in the genus *Planobacterium *, for which we propose the name *Planobacterium salipaludis* comb. nov.

*Planobacterium taklimakanense* is a rare example of agenus assignment – we had to consider whether or not to classify it as a member of the genus *Kaistella *. The AAI between *Planobacterium taklimakanense* NCTC 13490^T^ and CCUG 49689^T^, the type strain of *Kaistella koreensis* (the type species of the genus *Kaistella *) was intermediate at 75.1 %. The highest AAI that the type strain of *Planobacterium taklimakanense,* NCTC 13490^T^, shared with any other type strain was 76.25 % (*Kaistella haifensis*), and it had an AAI >76 % when compared to three of the type strains of species of the genus *Kaistella *, but it also had an AAI < 74 % when compared with the type strains of nine other species of the genus *Kaistella *. Several characteristics are shared among members of the proposed genus *Kaistella *, such as small genome size, presence of a carotenoid biosynthetic gene cluster (accessions numbers SNV33133–33186) including lycopene cyclase (SNV33175), absence of the *darA* gene (Accession number EFK37140 in *Chryseobacterium gleum *) and a corresponding absence of the flexirubin pigments that its encoded protein would produce, and a fatty acid composition with >10 % C_15 : 0_ anteiso. However, unlike the strains belonging to the genus *Kaistella *, *Planobacterium taklimakanense* lacks the *nosZ* gene (accession number KMQ71079 in *Kaistella koreensis *) and associated maturase genes necessary to produce nitrous oxide reductase. Core genome analysis placed the genus *Halpernia* between *Kaistella* and *Planobacterium *. Taken together, these findings are sufficient evidence to consider *Planobacterium* as a separate genus. The SBRL at CDC receives several specimens of this species every year for identification, and it should be recognised as a potential human pathogen.

Phylogenetic analysis of the 16S rRNA gene sequences (Fig. S1) of all species described as members of the genus *Chryseobacterium *, along with most other members of the family *Weeksellaceae* and *Flavobacterium aquatile* as the outgroup, places the recently published *Chryseobacterium reticulitermitis* near the base of the tree, near the genus *Soonwooa *. The effectively, but not validly, published *‘Chryseobacterium chengduensis’* clusters with *Daejongia ginsengosidivorans. Chryseobacterium frigidum* appears to be most closely related to *Cruicaptor ignavus*, but divergent phenotypically, both in cell shape and in major fatty acid composition. No whole-genome sequences exist for any of these species, so out of an abundance of caution we make no proposals for them at this time. In contrast, the 16S RNA gene species *Chryseobacterium salipaludis* is clustered with several fully sequenced strains of *Planobacterium taklimakanense *, and is phenotypically similar to them, providing sufficient evidence for our proposal of this species as *Planobacterium salipaludis* comb. nov. Similarly, both 16S rRNA gene sequence and phenotype of *Chryseobacterium marinum* were similar to those of the proposed species of the genus *Halpernia*, prompting us to designate this species as *Halpernia marina* comb. nov. All species of the genus *Kaistella* cluster together apart from any other genus, as do the species of the genera *Planobacterium* and *Halpernia* but there is some intermingling among the species of the genera *Epilithonimonas* and *Chryseobacterium *. In contrast, phylogenetic analysis of *rpoB* gene sequences (Fig. S3) provides a much clearer separation of the genera, and is in agreement with the core genome maximum likelihood analysis (Fig. 2) and with the genus assignments based on AAI.

Available phenotypic data from the literature for each of the 109 species listed as members of the genus *Chryseobacterium* in the LSPN as of September 2018, along with phenotypic data for two novel species described herein, has been tabulated (Table S2). Phenotypic differentiation of members of the family *Flavobacteriaceae* has long been recognised as difficult and unreliable, as variation within each species is substantial, and certain assays produce different results depending on the method used. For example, strains of members of the genus *Elizabethkingia* that were unable to grow on MacConkey agar when initially isolated gained the ability to do so after several passages [89]. Table S2 Despite these limitations, certain trends can be discerned and are discussed here. Because some data were not available, we describe the results as a fraction with the denominator representing the number of species in a particular genus that have data available.

Almost half of species of the genus *Chryseobacterium* tested (27 out of 66) degraded urea, but only one species of the genus *Kaistella* did, and none of the species of the genera *Halpernia* or *Epilithonimonas *. Most species of the genera *Chryseobacterium* (73/75), *Halpernia* (2/2), and *Epilithonimonas* (11/11) degraded aesculin, but almost half (6/13) of the species of the genus *Kaistella* did not. The only strains capable of growing on cetrimide agar were among the species of the genus *Chryseobacterium *, although only a minority of those (13 out of 31) were able to do so.

A few species of the genus *Chryseobacterium* (7/55) produced acid from mannitol while no species of the genera *Halpernia* (0/1), or *Kaistella* (0/11) did, and only a single species of the genus *Epilithonimonas* (1/8) produced acid from mannitol, but results varied for different laboratories. No species of the genus *Halpernia* (0/1) and only a single species of the genus *Kaistella* (1/11) produced acid from arabinose, but over 20 % of species of the genera *Chryseobacterium* (14/61) and *Epilithonimonas* (3/10) did. No species of the genus *Kaistella* (0/11) produced acid from trehalose, but half or more of species of the genera *Chryseobacterium* (33/52), *Epilithonimonas* (5/9), and *Halpernia* (1/2) did.

A novel phospholipid fatty acid, anteiso-C_17 : 2_ ω3,7, has been recently identified in the cell membrane of the type strain of *Halpernia frigidisoli* [90]. The fact that this fatty acid is predominant when the bacterium is grown at 0°C indicates its importance for cell membrane adaptation. All species of the genus *Halpernia* grew at 5 °C, and none grew at 42 °C. Both of the available genomes of members of the genus *Halpernia* contain proteorhodopsin and beta-carotene monoxygenase, neither of which were present in any genome from a member of the genera *Epilithonimonas, Chryseobacterium* or *Kaistella *.

Christensen *et al.* [38] recommended that multiple strains be used to describe a novel species, rather than a single individual strain, but publication of single-strain species descriptions continues. There have even been suggestions made that a single complete sequenced genome could be used as the type material for publication of a novel species, regardless of whether it can be cultured [37]. The advantage to this approach is that once a novel species is named, additional strains (particularly metagenome assembled genomes) can be assigned to it based on genome sequence similarity, thereby increasing the diversity of habitats known to be occupied by that particular species and improving our understanding of its biology. Unfortunately, single-strain species naming has led to a proliferation of species names, and there is a danger that if this trend continues, the list of validly published species names will become essentially a list of well-characterised named isolates. This could be avoided by limiting the naming of a species to those that have already been isolated more than once or encountered in more than one metagenomics sample. For these reasons, we have elected to release the genomes of several strains that are the sole known representative of their species, without naming them, in hopes of facilitating future collaborations.

## Emended description of the genus *Chryseobacterium* Vandamme *et al.* 1994

The description of the genus *Chryseobacterium* is as stated by Vandamme *et al.* [1] and emended by Kämpfer *et al.* [10], Wu *et al.* [91], and Chen *et al.* [92], with the following emendments: All species tested produce non-diffusible flexirubin type pigments. Most species do not reduce nitrate or nitrite, and most are not capable of growth at 42 °C. Most do not produce H_2_S. Tween-80 and starch are usually degraded. Acid production is common from glucose, maltose, and trehalose, but rare from lactose or mannitol. The DNA G+C content ranges from 28.8 to 49.3 mol%.

The type species is *Chryseobacterium gleum* Vandamme *et al*. (1994). Whole genome analysis of the type strain of each species produces AAI comparison values, using proteins that share 40 % amino acid identity and 50 % coverage length, of ≥76 % when compared with *Chryseobacterium gleum* strain F93^T^, and ≥74 % when compared with the type strain of each of the other species in the family. Species that have already been named as members of the genus *Chryseobacterium* but have not yet their type stain’s genome sequenced at this time and are not otherwise discussed in this manuscript can be assumed to remain in the genus *Chryseobacterium *.

## Emended description of *Chryseobacterium bernardetii* Holmes *et al*. 2013

The description is as given by Holmes *et al.* [12] and Kim *et al.* [64] with the following emendments: Different strains give different results for H_2_S production (lead acetate paper method) and nitrate reduction. The DNA G*+*C content of type strain G0229^T^ was calculated to be 36.3 mol%.

## Emended description of *Chryseobacterium indoltheticum* Campbell and Williams 1951 Vandamme *et al*. 1994

The description is as given by Campbell and Williams [93] and discussed by Bernardet *et al*. [94] with the following emendment: The DNA G+C content was 34.3 mol% for both strains G0141^T^ and G0211.

## Emended description of *Chryseobacterium lactis* Holmes *et al*. 2013

The description is as given by Holmes *et al.* [12] with the following emendment: The DNA G*+*C content of the type strain KC1864^T^ was calculated to be 36.1 mol%.

## Emended description of *Chryseobacterium nakagawai* Holmes *et al*. 2013

The description is as given by Holmes *et al.* [12] with the following emendments: strains are positive for acid production (in ammonium salt medium) from glycerol, aesculin hydrolysis, growth on cetrimide agar, hydrolysis of Tween 80, and utilisation of citrate (Christensen’s medium). The DNA G*+*C content of the type strain, G0041^T^, was calculated to be 35.4 mol%.

## Emended description of *Chryseobacterium shandongense* Yang *et al*. 2015

The description is as given by Yang *et al.* [86], with the following emendments: Acid is produced from glucose, ethanol and maltose but not from adonitol, dulcitol, glycerol, inositol, lactose, mannitol, raffinose, salicin, sorbitol or sucrose. Hydrolysis of starch, gelatin and DNA varies between strains, as does growth on MacConkey agar.

## Emended description of the genus *Epilithonimonas* O'Sullivan *et al*. 2006

The description is as given by O'Sullivan *et al.* [13] but with the following emendments:

Colonies are circular, entire and convex and may be non-pigmented or yellow to bright orange. Cannot produce acid from mannitol. Does not reduce nitrite, hydrolyze urea or produce hydrogen sulphide or arginine dihydrolase but does degrade aesculin. Will not grow on cetrimide agar, and cannot tolerate 3 % NaCl. The DNA G+C content is between 33.3 and 39.2 mol%.

The type species is *Epilithonimonas tenax *. Whole genome analysis of the type strain of each species produces AAI comparison values, using proteins that share 40 % amino acid identity and 50 % coverage length, of ≥76 % when compared with *Epilithonimonas tenax* strain EP105^T^, and ≥74 % when compared with the type strain of each of the other species in the genus.

## Description of *Epilithonimonas arachidiradicis* comb. nov.

*Epilithonimonas arachidiradicis* (a.ra.chi.di.ra′di.cis. N.L. fem. n. *Arachis - idis* the generic name of the peanut plant; L. fem. n. *radix - icis* root; N.L. gen. n. *arachidiradicis* of the root of *Arachis*).

Basonym: *Chryseobacterium arachidiradicis* Kämpfer *et al*. 2015

The description is as given by Kämpfer *et al.* [95]. The type strain is 91A-612^T^ = LMG 27814^T^ = CCM 8490^T^= CIP 110647^T^.

## Description of *Epilithonimonas bovis* comb. nov.

*Epilithonimonas bovis* (bo′vis. L. gen. n. *bovis* of a cow, referring to the isolation from raw cow's milk).

Basonym: *Chryseobacterium bovis* Hantsis-Zacharov *et al*. 2008

The description is as given by Hantsis-Zacharov *et al.* [96]. The type strain is H9^T^=DSM 19482^T^=LMG 24227^T^.

## Description of *Epilithonimonas caeni* comb. nov.

*Epilithonimonas caeni* (cae′ni. L. gen. n. *caeni* of sludge).

Basonym: *Chryseobacterium caeni* Quan *et al.* 2007

The description is as given by Quan *et al.* [97] as emended by Hahnke *et al.* [18]. The type strain is N4^T^ = CCBAU 10201^T^=DSM 17710^T^=KCTC 12506^T^.

## Description of *Epilithonimonas hispanica* comb. nov.

*Epilithonimonas hispanica* (his.pa′ni.ca. L. fem. adj. *hispanica* from Spain).

Basonym: *Chryseobacterium hispanicum* Gallego *et al.* 2006

The description is as given Gallego *et al.* [98]. The type strain is VP48^T^=CECT 7129^T^=CCM 7359^T^=JCM 13554^T^.

## Description of *Epilithonimonas hominis* comb. nov.

*Epilithonimonas hominis* (ho′mi.nis. L. gen. n. *hominis* of a man, of a human being, named as such because most of the known isolates at the time of description were of human origin).

Basonym: *Chryseobacterium hominis* Vaneechoutte *et al.* 2007

The description is as given by Vaneechoutte *et al.* [99]. The type strain is NF802^T^=CCUG 52711^T^=CIP 109415^T^.

## Description of *Epilithonimonas hungarica* comb. nov.

*Epilithonimonas hungarica* (hun.ga′ri.ca. N.L. fem. adj. *hungarica* from Hungary, referring to the country from which the type strain was isolated).

Basonym: *Chryseobacterium hungaricum* Szoboszlay *et al.* 2008

The description is as given by Szoboszlay *et al.* [100]. The type strain is CHB-20p^T^=NCAIM B2269^T^=DSM 19684^T^.

## Description of *Epilithonimonas mollis* comb. nov.

*Epilithonimonas mollis* (mol′lis. L. fem. adj. *mollis* pliant, sensitive, referring to the sensitivity to antibiotics).

Basonym: *Chryseobacterium molle* Herzog *et al.* 2008.

The description is the same as given by Herzog *et al.* [101]. The type strain is DW3^T^=DSM 18016^T^=CCUG 52547^T^.

## Description of *Epilithonimonas pallida* comb. nov.

*Epilithonimonas pallida* (pal′li.da. L. fem. adj. *pallida* pale).

Basonym: *Chryseobacterium pallidum* Herzog *et al.* 2008

The description is the same as given by Herzog *et al.* [101]. The type strain is 26-3St2b^T^=DSM 18015^T^=CCUG 52548^T^.

## Description of *Epilithonimonas zeae* comb. nov.

*Epilithonimonas zeae* (ze′ae. L. gen. n. *zeae*, of spelt, of *Zea mays*).

Basonym: *Chryseobacterium zeae* Kämpfer *et al.* 2014

The description is as given by Kämpfer *et al.* [102]. The type strain is JM-1085^T^=LMG 27809^T^=CCM 8491^T^.

## Description of *Epilithonimonas vandammei* sp. nov.

*Epilithonimonas vandammei* (van.dam′me.i. N.L. gen. masc. n. *vandammei* named in honor of Peter Vandamme in recognition of his many contributions to the study of the genus *Chryseobacterium* and related genera).

Cells are Gram-stain-negative. Colonies are circular, convex, entire, opaque, shiny, smooth and usually yellow-pigmented. Positive for acid production (in ammonium salt medium) from glucose and maltose, aesculin hydrolysis, catalase production, cytochrome oxidase production, growth at 37 °C, at room temperature (18–22 °C) and on β-hydroxybutyrate. Negative for acid production (in ammonium salt medium) from adonitol, arabinose, cellobiose, dulcitol, inositol, lactose, raffinose, salicin, sorbitol, trehalose and xylose, arginine dihydrolase production, fluorescence on King’s B medium, gas production from glucose in peptone water medium, gluconate oxidation, growth at 5 °C and at 42 °C and on cetrimide agar, hydrolysis of tyrosine, H_2_S production (by both lead acetate paper and triple sugar iron agar methods), KCN tolerance, lecithinase production, lipid inclusions after growth on β-hydroxybutyrate, lysine decarboxylase production, malonate utilisation, motility (hanging drop preparation at both 37 °C and room temperature), ornithine decarboxylase production, phenylalanine deamination, production of β-galactosidase (ONPG test), reduction of 0.4 % (w/v) selenite, urease production, utilisation of citrate (Christensen’s and Simmons’ media) and 3-ketolactose production.

The type strain is F5649^T^=CCUG 73498^T^=CIP 111693^T^ and was derived from a human clinical testicle isolate from Iowa, USA, in 1984. The DNA G+C content of the type strain has been calculated from its genome sequence to be 37 mol%.

## Description of *Halpernia* gen. nov.

*Halpernia* (Hal.per′ni.a. N.L. fem. n. *Halpernia* named after Malka Halpern, Professor at University of Haifa, Haifa, Israel, in recognition of her many contributions to the study of the genus *Chryseobacterium* and related genera.)

Cells are aerobic. Colonies are yellow-pigmented. Capable of growth on 3 % NaCl. Grows at 5 and 25 °C. Starch is hydrolyzed. Does not degrade urea. Positive for acid production from cellobiose, glucose and lactose; weakly positive for acid production from salicin. Whole-genome analysis of the type strain of each species produces AAI comparison values, using proteins that share 40 % amino acid identity and 50 % coverage length, of ≥76 % when compared with *Halpernia frigidisoli* strain PB4^T^, and ≥74 % when compared with the type strain of each of the other species in the genus. The DNA G+C content is 33.7–34 mol%.

The type species is *Halpernia frigidisoli.*

## Description of *Halpernia frigidisoli* comb. nov.

*Halpernia frigidisoli* (fri.gi.di.so′li. L. adj. *frigidus* cold, cool, chilled; L. neut. n. *solum* soil; N.L. gen. n. *frigidisoli* pertaining to cold soil, as the strain was isolated from a cold Antarctic soil).

Basonym: *Chryseobacterium frigidisoli* Bajerski *et al.* 2013

The description is as given by Bajerski *et al*. [103]. The type strain is PB4^T^=DSM 26000^T^=LMG 27025^T^.

## Description of *Halpernia humi* comb. nov.

*Halpernia humi* (hu′mi. L. gen. n. *humi* of earth, soil).

Basonym: *Chryseobacterium humi* Pires *et al.* 2010

The description is as given by Pires *et al.* [104]. The type strain is ECP37^T^=LMG 24684^T^=NBRC 104927^T^.

## Description of *Halpernia marina* comb. nov.

*Halpernia marina* (ma.ri′na. L. fem. adj. *marina* of the sea, marine).

Basonyms: *Sejongia marina* Lee *et al.* 2007, *Chryseobacterium marinum* Kämpfer *et al.* 2009

The description is as given by Lee *et al.* [8] and as emended by Kämpfer *et al.* [9] . The type strain is IMCC3228^T^ (=KCCM 42689^T^=NBRC 103143^T^).

## Emended description of the genus *Kaistella* Kim *et al.* 2004

The description is as given by Kim *﻿et al.* [5], but is emended as follows:

All strains grow at room temperature; Carotenoid pigments are usually produced. Colonies may be opaque, translucent or transparent. Catalase and oxidase are usually, but not always, positive, and arginine dihydrolase and β-galactosidase are usually, but not always, negative. Aesculin hydrolysis is present in some species. Nitrite is not reduced, and most species do not reduce nitrate. Does not grow on cetrimide agar. Acid production from mannitol, trehalose and xylose is negative, acid production from glucose varies between species. The DNA base composition ranges from 31.3 to 41.6 mol% G+C.

The type species is *Kaistella koreensis *. Whole-genome analysis of the type strain of each species produces AAI comparison values, using proteins that share 40 % amino acid identity and 50 % coverage length, of ≥76 % when compared with *Kaistella koreensis* strain Chj707 ^T^, and ≥74 % when compared with the type strain of each of the other species in the genus.

## Description of *Kaistella anthropi* comb. nov.

*Kaistella anthropi* (an′thro.pi. Gr. n. *anthropos*, a human being; N.L. gen. n. *anthropi*, of a human being, since all strains so far recovered are from human clinical specimens).

Basonym: *Chryseobacterium anthropi* Kämpfer *et al.* 2009

The description is as given by Kämpfer *et al.* [10]. The type strain is NF 1366^T^=CCUG 52764^T^=CIP 109762^T^.

## Description of *Kaistella antarctica* comb. nov.

*Kaistella antarctica* (ant.arc′ti.ca. L. fem. adj. *antarctica* southern, named after Antarctica, the geographical origin of the type strain).

Basonyms: *Sejongia antarctica* Yi *et al.* 2005, *Chryseobacterium antarcticum* Kämpfer *et al.* 2009

The description is as given by Yi *et al*. [7] and emended by Kämpfer et al. [9] and Hahnke, et al. [18]. The type strain is AT1013^T^=IMSNU 14040^T^=KCTC 12225^T^=JCM 12381^T^.

## Description of *Kaistella carnis* comb. nov.

*Kaistella carnis (*car′nis. L. fem. n. *carnis*, of flesh)

Basonym: *Chryseobacterium carnis* Holmes *et al.* 2013

The description is as given by Holmes *et al.* [12], with the following emendment: The type strain is G0081^T^=NCTC 13525^T^=CCUG 60559^T^=CL88/78^T^=Hayes B19/1^T^. The DNA G*+*C content of type strain G0081 was calculated to be 36.4 mol%.

## Description of *Kaistella chaponensis* comb. nov.

*Kaistella chaponensis* (cha.po.nen′sis. N.L. fem. adj. *chaponensis* pertaining to Lake Chapo, Chile, from which the Atlantic salmon harbouring the original two isolates was obtained)

Basonym: *Chryseobacterium chaponense* Kämpfer *et al.* 2011

The description is as given by Kämpfer *et al.* [105]. The type strain is Sa 1147–06^T^=DSM 23145^T^=CCM 7737^T^.

## Description of *Kaistella daneshvariae* sp. nov.

*Kaistella daneshvariae* (da.nesh.va′ri.ae. N.L. gen. fem. n. *daneshvariae* named in honor of Maryam Daneshvar, for her many contributions to the description and characterization of strains from CDC’s collection of clinical bacterial isolates).

Cells are Gram-stain-negative, rod shaped, aerobic and non-motile. Colonies on blood agar with 5 % rabbit’s blood are yellow-pigmented, convex and smooth with no lysis and may appear mucoid or runny. Growth occurs at 25 and 35° but not at 42 °C. Does not require NaCl or tolerate high (6 %) NaCl. Does not grow on MacConkey’s, citrate or cetrimide agars. Catalase- and oxidase-positive, and positive for indole production. Does not hydrolyze urea, aesculin or gelatin, and does not reduce nitrate. Produces H_2_S on lead acetate paper but not triple sugar iron agar. Acid production may occur from glucose, but not from d-xylose, mannitol, lactose, sucrose or maltose.

The type strain is H3001^T^ (=CCUG 73276^T^=CIP 111694^T^), which was isolated from the peritoneal cavity of a patient in the state of New York, USA. The DNA G+C content of the type strain is 39.9 %. A second reference strain is H3056 (=CCUG 73499), which was isolated as a blood culture from a patient in New Mexico, USA.

## Description of *Kaistella haifensis* comb. nov.

*Kaistella haifensis* (hai.fen′sis. N.L. fem. adj. *haifensis* pertaining to Haifa, the name of the university (University of Haifa) where the first isolates were studied.)

Basonym: *Chryseobacterium haifense* Hantsis-Zacharov and Halpern 2007

The description is the same as for *Chryseobacterium haifense* [85]. The type strain is H38^T^=DSM 19056^T^=LMG 24029^T^.

## Description of *Kaistella jeonii* comb. nov.

*Kaistella jeonii* (jeo′ni.i. N.L. gen. n. *jeonii* named in honour of the late Jae Gyu Jeon, who devoted his life to polar research.)

Basonyms: *Sejongia jeonii* Yi *et al.* 2005, *Chryseobacterium jeonii* Kämpfer *et al.* 2009

The description is as given by Yi *et al.* [7] as emended by Kämpfer *et al.*[9] . The type strain is AT1047^T^=IMSNU 14049^T^=KCTC 12226^T^=JCM 12382^T^.

## Description of *Kaistella montana* comb. nov.

*Kaistella montana* sp. nov. (mon.ta′na. L. fem. adj. *montana* living in the mountains).

Basonym: *Chryseobacterium montanum* Guo *et al.* 2016

The description is as given by Guo *et al*. [106]. The type strain is WG4^T^=KCTC 52204^T^=CCTCC AB 2016058^T^.

## Description of *Kaistella palustris* comb. nov.

*Kaistella palustris* (pa.lus′tris. L. fem. adj. *palustris* pertaining to a marsh)

Basonym: *Chryseobacterium palustre* Pires *et al.* 2010

The description is as given by Pires *et al*. [104] as emended by Hahnke, *et al.* [18]. The type strain is 3A10^T^ (=LMG 24685^T^=NBRC 104928^T^).

## Description of *Kaistella solincola* comb. nov.

*Kaistella solincola* (sol.in′co.la. L. neut. n. *solum* soil; L. masc. or fem. n. *incola* an inhabitant; N.L. n. *solincola* an inhabitant of soil).

Basonym : *Chryseobacterium solincola* Benmalek *et al*. 2010

The description is as given by Benmalek *et al*. [107] as emended by Hahnke, *et al.* [18]. The type strain is 1YB-R12^T^=DSM 22468^T^=CCUG 55604^T^.

## Description of *Kaistella treverensis* comb. nov.

*Kaistella treverensis* (tre.ve.ren′sis. N.L. fem. adj. *treverensis*, pertaining to Augusta Trevirorum, the Latin name of Treves [Trier, West Germany], the city from which the strain was sent for identification).

Basonym: *Chryseobacterium treverense* Yassin *et al.* 2010

The description is as given by Yassin *et al*. [108]. The type strain is IMMIB L-1519^T^=CCUG 57657^T^=DSM 22251^T^.

## Description of *Kaistella yonginensis* comb. nov.

*Kaistella yonginensis* (yon.gi.nen′sis. N.L. fem. adj. *yonginensis* of or belonging to Yongin, Korea, from where the type strain was isolated).

Basonym: *Chryseobacterium yonginense* Joung and Joh, 2011

The description is as given by Joung and Joh [109]. The type strain is HMD1043^T^=KCTC 22744^T^=CECT 7547^T^.

## Emended description of the genus *Planobacterium* Peng *et al*. 2009

The description is as given by Peng e*﻿t al.* [11].

The type species of the genus is *Planobacterium taklimakanense* Peng *et al.* (2009). Despite the naming of the genus based on motility of the first isolate, motility has not been observed in subsequent isolates. The predominant cellular fatty acids are iso-C_15 : 0_ and anteiso-C_15 : 0_. Whole-genome analysis of the type strain of each species produces AAI comparison values, using proteins that share 40 % amino acid identity and 50 % coverage length, of ≥76 % when compared with *Planobacterium taklimakanense* strain X-65^T^, and ≥74 % when compared with the type strain of each of the other species in the genus.

## Emended description of *Planobacterium taklimakanense* Peng *et al.* 2009

The description is as given by Peng *﻿et al.* [11] and Kim *et al*. [64] but with the following amendment: The DNA G+C content of strain NCTC 13490^T^ is 40.4 mol% based on WGS, and reference strains H4753 and F9257 have DNA G+C contents of 40.8 mol% and 40.7 mol%, respectively.

## Description of *Planobacterium salipaludis* comb. nov.

*Planobacterium salipaludis* (sa.li.pa.lu′dis. L. n. *sal*, salt; L. gen. n. *paludis,* of a swamp; N.L. gen. n. *salipaludis*, of a salt marsh)

Basonym: *Chryseobacterium salipaludis* Divyasree *et al.* 2018

The description is as given by Divyasree *et al*. [110]. The type strain is JC490^T^ (KCTC 52835^T^=LMG 30048^T^).

## Supplementary Data

Supplementary material 1

Supplementary material 2

Supplementary material 3
