# Supplementary material for: Division of the genus Chryseobacterium: Observation of discontinuities in amino acid identity values, a possible consequence of major extinction events, guides transfer of nine species to the genus Epilithonimonas, eleven species to the genus Kaistella, and three species to the genus Halpernia gen. nov., with description of Kaistella daneshvariae sp. nov. and Epilithonimonas vandammei sp. nov. derived from clinical specimens
Source: Int J Syst Evol Microbiol. 2020 Jan 2;70(8):4432–50. doi: 10.1099/ijsem.0.003935 (PMC7660247; doi:10.1099/ijsem.0.003935)
Supplement: Supplementary material 1 [file ijsem-70-4432-s001.pdf]

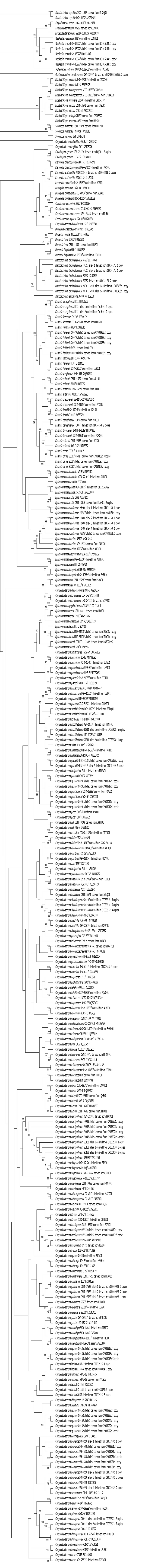

Figure S1: 16S rRNA gene Neighbour-Joining phylogenetic tree. Sequences that were located using BLAST and extracted from whole genome files are indicated by reference to the genbank accession number or SRA file from which they were derived, and those extracted from complete circular genomes are additionally annotated with the number of copies of that specific gene variant identified within the genome. All available genomes were inspected for the presence of a 16S rRNA gene; the absence of a 16S rRNA gene on the phylogenetic tree when a genome was available is indicative of its complete (eg, *Chryseobacterium bovis*) or partial (eg, "*Chryseobacterium mucoviscidosis*") absence in the genome assembly. Species that are indicated by AAI to be generically misclassified, but for which no proposals are made in this paper, are indicated by brackets. The percentage of replicate trees in which the associated taxa clustered together in the bootstrap test (1000 replicates) is shown next to the branches. The evolutionary distances were computed using the Jukes-Cantor method and are in the units of the number of base substitutions per site. The analysis involved 301 nucleotide sequences. All positions containing gaps and missing data were eliminated. There were a total of 1067 positions in the final dataset.

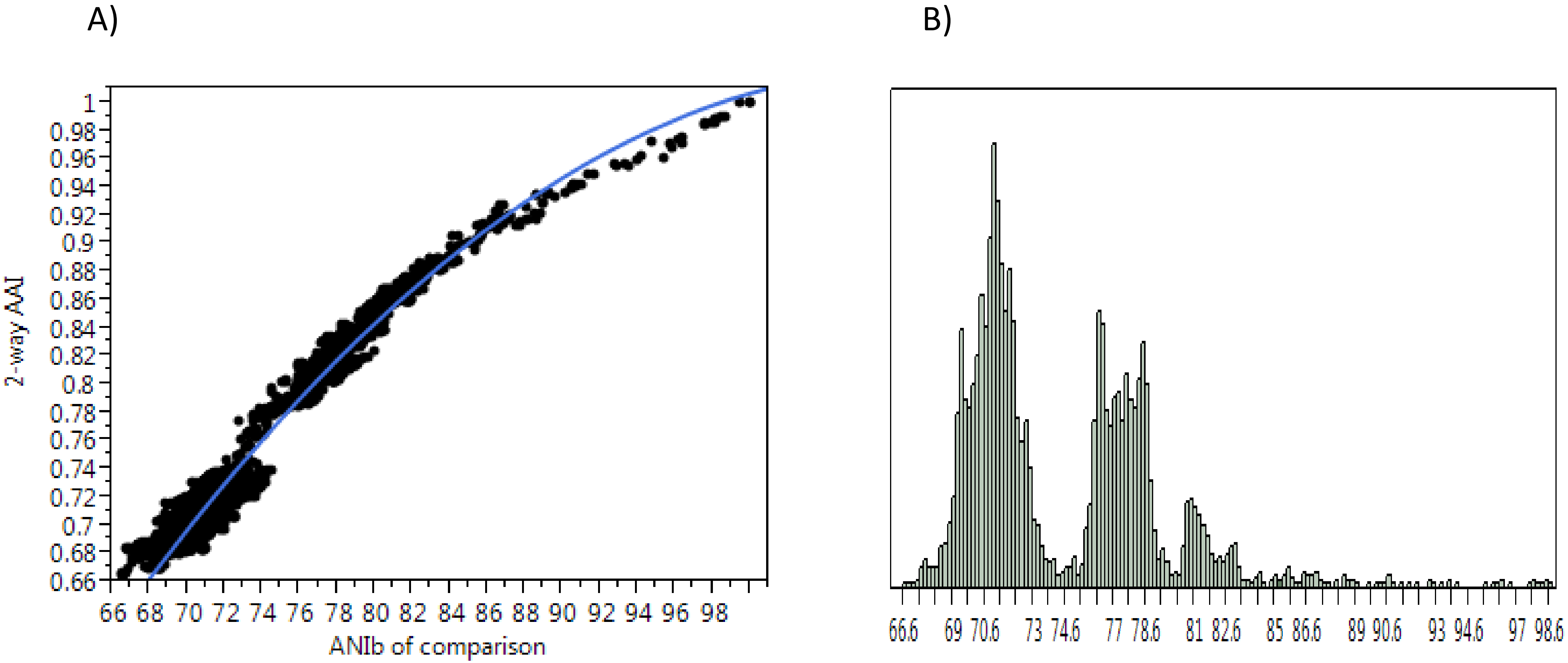

Figure S2: a) Bivariate fit of ANIb and AAI calculated for each strain comparison. A quadratic fit predicts AAI from ANIb. b) Distribution of ANIb values shows that few strain comparisons have a ANIb between 72.6% and 74%, which corresponds to the AAI region between 74% and 76% noted in Figure 1.

Figure S3: Timing of major extinction events and ANI of strains diverging afterwards. The distribution of ANI results from strain comparisons between type strains of Chryseobacterium species and their closest relatives can be superimposed onto a timeline showing how many millions of years ago the three most recent major extinction events occurred. This analysis is restricted to strains belonging to different species, so no peak is seen that would represent diversion occurring after the K-Pg event. It is however quite obvious that strains diverging after this event would have an ANI value of 95% or greater, meeting the accepted species definition.

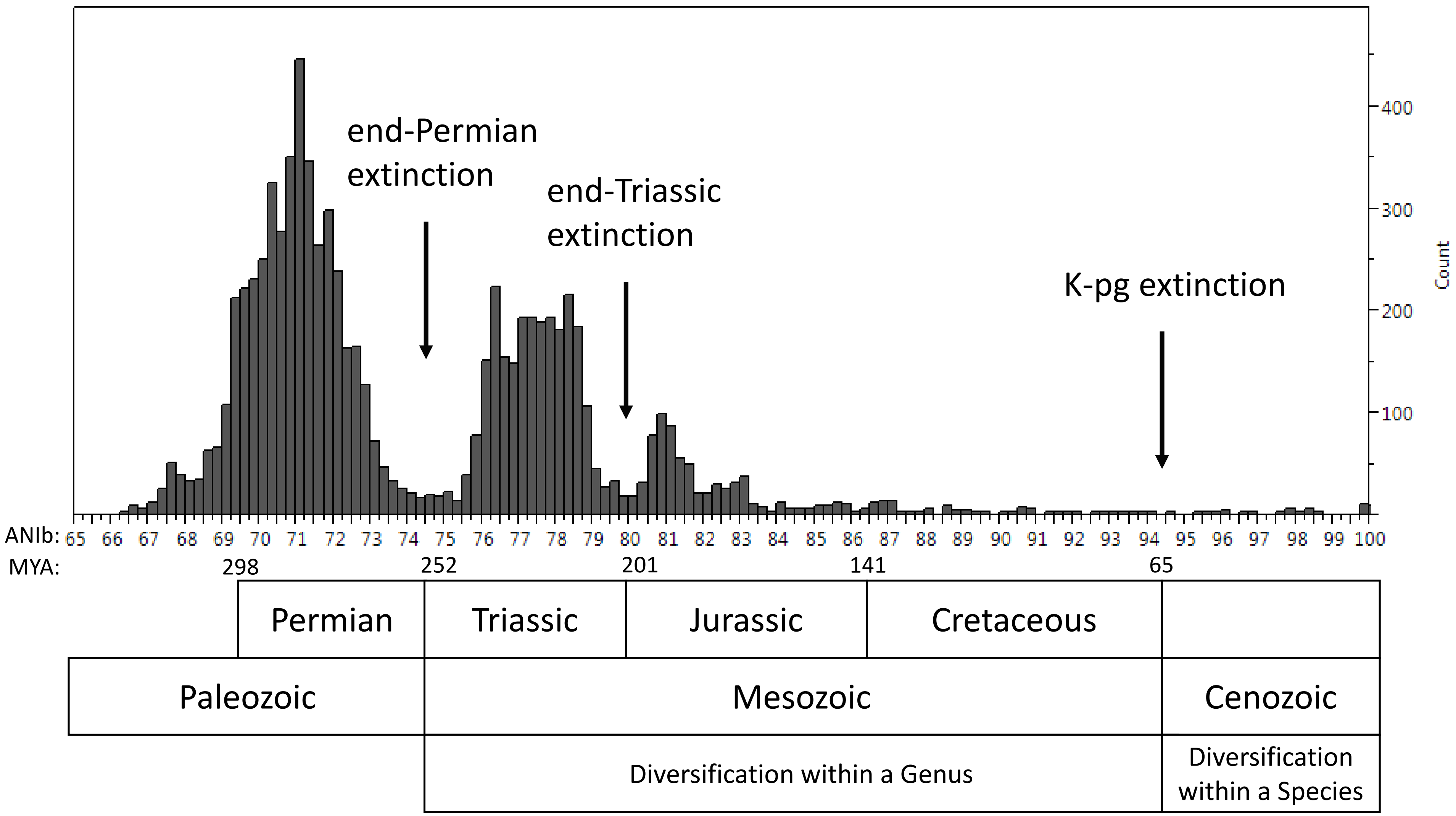

Figure S4: Maximum Likelihood rpoB tree. The percentage of trees in which the associated taxa clustered together is shown next to the branches. The tree is drawn to scale, with branch lengths measured in the number of substitutions per site. The analysis involved 122 nucleotide sequences and there were a total of 1622 positions in the final dataset. Evolutionary analyses were conducted in MEGA X and based on the Jukes-Cantor model.

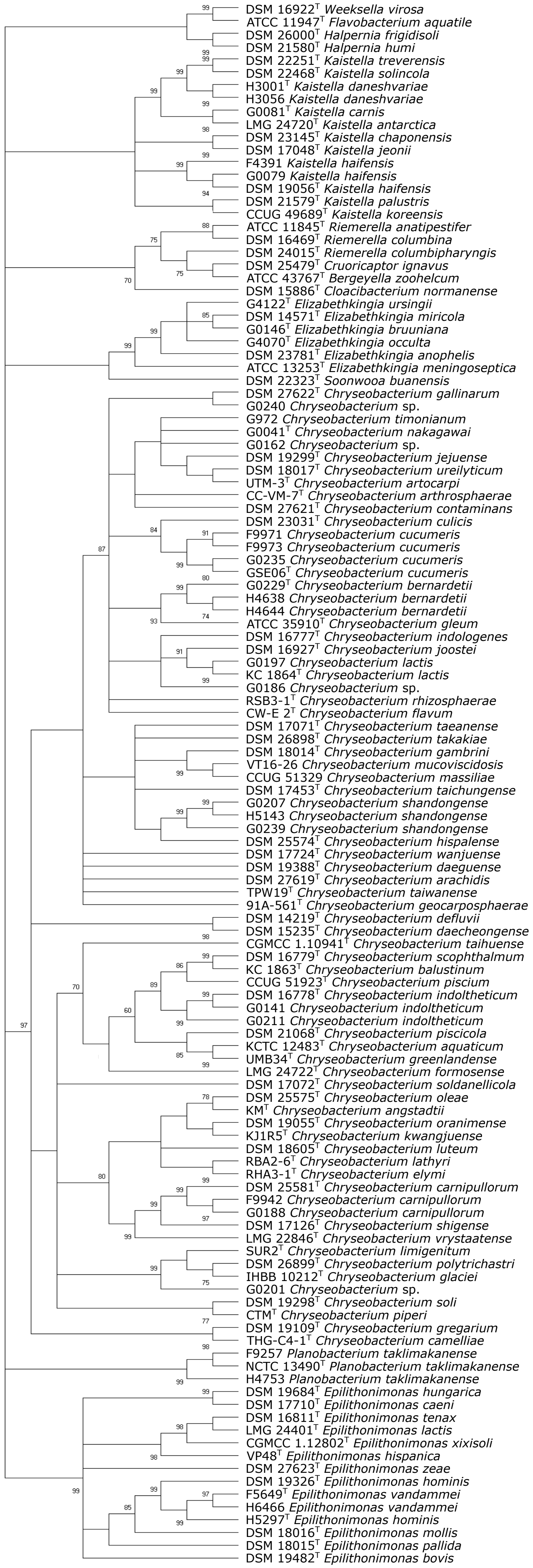

Click to open Supplemental Table 1: Accession numbers of public-domain genomes used in this study.

Click to open Supplemental Table 2: Phenoytypic data for *Chryseobacterium* species. For the purposes of this summary, a result reported as “weakly positive” is considered as a positive result.
